# Asymptomatic COVID-19 Adult Outpatients identified as Significant Viable SARS-CoV-2 Shedders

**DOI:** 10.1038/s41598-021-00142-8

**Published:** 2021-10-18

**Authors:** Marie Glenet, Anne-Laure Lebreil, Laetitia Heng, Yohan N’Guyen, Ittah Meyer, Laurent Andreoletti

**Affiliations:** 1 Cardiovir EA-4684, Unité de Recherche, Université de Reims Champagne Ardenne, Reims, France; 2 Service de Médecine Interne, Immunologie clinique et maladies infectieuses, Centre Hospitalier Universitaire Reims, 51097 Reims, France; 3 Unilabs BioCT, Private Laboratory Group, Château-Thierry, France; 4 Laboratoire de Virologie Médicale et Moléculaire, CHU Reims, Hôpital Robert Debré, Avenue du Général Koenig, 51092 Reims Cedex, France

**Keywords:** Infectious diseases, Infection

## Abstract

Differential kinetics of RNA loads and infectious viral levels in the upper respiratory tract between asymptomatic and symptomatic SARS-CoV-2 infected adult outpatients remain unclear limiting recommendations that may guide clinical management, infection control measures and occupational health decisions. In the present investigation, 496 (2.8%) of 17,911 French adult outpatients were positive for an upper respiratory tract SARS-CoV-2 RNA detection by a quantitative RT-PCR assay, of which 180 (36.3%) were COVID-19 asymptomatic. Of these adult asymptomatic viral shedders, 75% had mean to high RNA viral loads (Ct values < 30) which median value was significantly higher than that observed in symptomatic subjects (*P* = 0.029), and 50.6% were positive by cell culture assays of their upper respiratory tract specimens. Our findings indicate that COVID-19 asymptomatic adult outpatients are significant viable SARS-CoV-2 shedders in their upper respiratory tract playing a major potential role as SARS-CoV-2 transmitters in various epidemiological transmission chains, promoting COVID-19 resurgence in populations.

## Introduction

In December 2019 a novel Coronavirus, referenced SARS-CoV-2, was identified as the causative agent of a new respiratory illness designated as COVID-19. Despite of the announced containment measures taken by China, COVID-19 quickly became pandemic and was declared as a Global Health Emergency by World Health Organization. The full clinical spectrum of COVID-19 ranges from mild, self-limiting respiratory tract illness to severe progressive pneumonia, acute severe respiratory distress syndrome, multi-organ failure and death^1^. Rapid investigations in basic virology made it possible to obtain the complete sequence of the RNA viral genome and thus to develop specific and sensitive molecular tests for a reliable detection of this new emerging virus in human respiratory samples^2^. From January 2020 to the present time, the reference biological diagnosis of COVID-19 is based on detection of SARS-CoV-2 RNA genome by RT-qPCR testing of a nasopharyngeal swab^3^. Using some of referenced assays the SARS-CoV-2 RNA loads can be quantitatively estimated by threshold cycle (Ct) which values lower than 30 may be associated to infectious virus detectable by cell culture assays in the respiratory tract of symptomatic or asymptomatic carriers^4,5^. To date, differential kinetics of RNA loads and infectious viral levels between asymptomatic and symptomatic SARS-CoV-2 infected adult outpatients remain unclear limiting recommendations about the use of respiratory tract Ct values that may guide clinical management, infection control measures and occupational health decisions^3,4^. In the present study, we assessed the distribution of viral loads estimated by Ct values according to the presence of reported symptoms and to the delay between the respiratory sample and the onset of symptoms in study outpatients covering a large age-range. Moreover, among identified COVID-19 asymptomatic patients with high viral RNA loads, we investigated the presence of infectious SARS-CoV-2 particles in their upper respiratory tract specimens by cell culture assays.

## Patients and methods

Following to the first national COVID-19 lockdown occurring from 11 March to 13 May of 2020, France initiated a massive viral screening strategy authorizing any person with or without a medical prescription to perform an RT-qPCR detection test for SARS-CoV-2, resulting in the performance of 720–750,000 tests per week on an outpatient basis (French national public health data). From the 4th June to 20th September of 2020 in Champagne-Ardenne area (Norther east of France) we performed a SARS-CoV-2 RT-qPCR assay in trans nasal respiratory samples taken from 17,911 out patients [11,820 women/6,091 men; mean age (± SD): 41.79 ± 22.52 years (0–99 years)]. For each study patient basic demographic data and reports of COVID-19 symptoms and the time delay between the sampling and the onset of reported symptoms were recovered and registered by a medical pathologist (MD) at the time of the trans-nasal sample. The Institutional Ethics Committee (IEC), University hospital of Reims (Grand-Est, France) waived the requirement of informed consent and approved the study. Our protocols and investigations conformed to the principles outlined in the Declaration of Helsinki for use of human tissue or subjects.

Our quantitative real-time RT-PCR assay was based on the co-detection of two different RNA-dependent RNA polymerase (RdRp) viral gene target sequences (IP2 and IP4 respectively) in trans nasal respiratory samples as described previously^2^.

Cell culture assays for the detection of infectious SARS-CoV-2 in respiratory specimens were performed using Vero E6 cells with a procedure adapted from the CDC protocol^6^. Briefly, 300 µl of Vero cell suspension at concentration of 6 × 10^5^ cells per ml were mixed to 200 µl of clinical specimen onto a 24-well tissue culture plate. The inoculated cultures were grown at 37 °C in incubator at 5% CO_2_ for 96 h and monitored daily to examine the presence of specific cytopathic effects^6^. Following 96 h of cell culture, we determined the presence of infectious replicative particle in 200 µl of supernatants of cell culture wells using a referenced RT-PCR assays targeting RdRp gene^6^. Fold of RNA viral loads increase per ml of cell culture supernatants was estimated for each tested respiratory specimen using comparison of Ct values obtained from total RNA extracted from 100 µl of clinical sample and those obtained from 100 µl of supernatants at 96 h post-inoculation. Fold values greater than 1 were considered as representative of a viral RNA replication activity related to an infection of cultured cells by a viable viral strain for each tested respiratory specimen. Finally, at 96 h post infection of cultured Vero cells, a classical lysis range titration was performed to determine a viral titer (PFU/mL) for each tested respiratory specimen.

Quantitative variables were all compared using the Mann Whitney U test or two-way ANOVA test and a *P* value < 0.05 was considered statistically significant. Data were also represented as standard curve to regression sigmoidal with 95% confidence. All statistical analyses were performed using GraphPad Prism 8 (GraphPad) and SAS version 9.4 (SAS Institute Inc.).

### Ethical approval

The Institutional Ethics Committee (IEC), University hospital of Reims (Grand-Est, France) waived the requirement of informed consent and approved the study. Patients did not provide individual consent because of the retrospective and non-interventional nature of this study, in accordance with French legislation. No patient had previously raised an opposition to the use of their medical records data or the samples collected at the time of diagnosis. Data confidentiality was preserved during this internal study (Reims University Hospital GDPR register number RMR004-061120), and collection of biological specimens was registered in French Ministry of Higher Education and Research as number DC-2020-4052.

## Results

**Figure 1:**
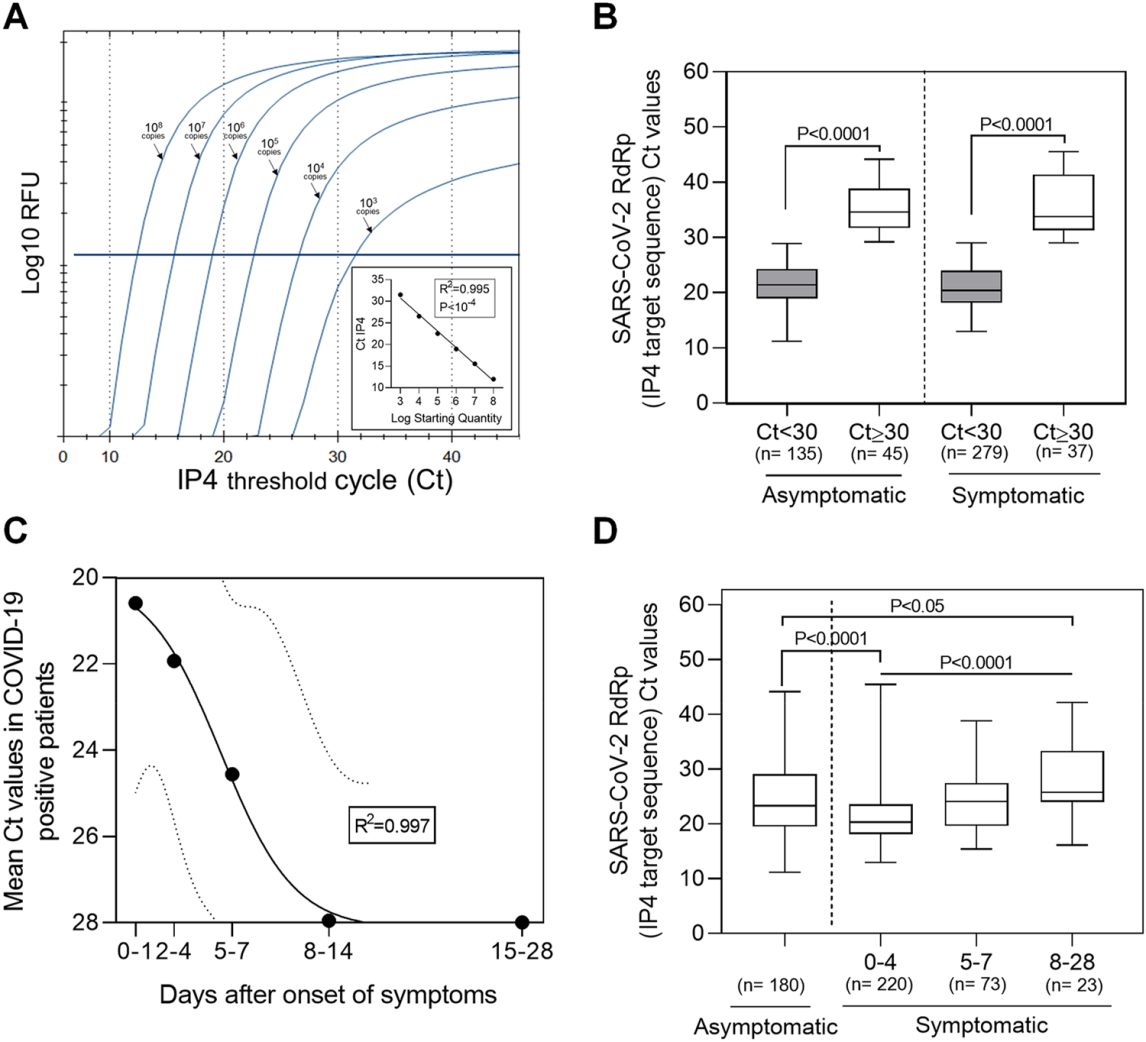
Distribution of viral loads estimated by SARS-CoV-2 RT-qPCR according to the presence of clinical symptoms and to the delay between the respiratory sample and the onset of symptoms in outpatients. (**A**) Cycle threshold (Ct) values of RNA dependent RNA polymerase (RdRp) target sequence (IP4) on SARS-CoV-2 RNA genome after serial ten-fold dilutions of synthetic SARS-CoV-2 RNA transcripts ranging from 10^8^ to 10^3^ copies. These positive controls for used for real-time RT-qPCR were synthetic RNA derived from cDNA plasmid of the BetaCoV_Wuhan_WIV04_2019 strain (EPI_ISL_402124). A significate correlation (R^2^ = 0.995, *P* < 10^–4^) was displayed between Ct values of IP4 sequence of SARS-CoV-2 and log_10_ starting quantity of SARS-CoV-2 RNA copies: this curve was used in each RT-qPCR serial analyses to validate our quantitative assay and to check that obtained Ct values inversely reflected viral load levels. (**B**) and (**D**) Boxes denote interquartile ranges, and horizontal bars denote median CT values of IP4 sequence among patients asymptomatic or symptomatic. Whiskers IP4 Ct values denote the maximum and minimum values below or above the median. (**B**) The shaded boxes indicate IP4 Ct values < 30 among asymptomatic (n = 135) or symptomatic (n = 279) patients whereas white boxes indicate IP4 Ct values ≥ 30 among asymptomatic (n = 45) or symptomatic (n = 37) patients (*P* < 0.0001). (**C**) Correlation of means Ct values in COVID-19 positive patients and days after onset of symptoms. The outer dotted lines are 95% confidence interval. Data represent standard curve to regression sigmoidal with a goodness of fit (R^2^ = 0.997). (**D**) Comparison of IP4 Ct values between asymptomatic outpatients (n = 180) and symptomatic sub-groups of outpatients stratified according to the delay between the respiratory sample and the onset of symptoms; 0–4 (n = 220), 5–7 (n = 73) and 8–28 (n = 23) days after the apparition of clinical symptoms (*P* < 0.0001). *Data represent the median* ± SD. *: P < *0.05; according to ANOVA test. Data represent the median* ± SD. *: P < 0.05; ****: P < *0.0001; according to ANOVA test.*

Quantitative cycle threshold (Ct) values of RdRp (RNA dependent RNA polymerase) RNA target sequence (IP4) of SARS-CoV-2 were obtained after serial ten-fold dilutions of synthetic SARS-CoV-2 RNA transcripts ranging from 10^8^ to 10^3^ copies. These positive controls for used for real-time RT-qPCR were synthetic RNA derived from cDNA plasmid of the BetaCoV_Wuhan_WIV04_2019 strain (EPI_ISL_402124). A significant correlation (R^2^ = 0.995, *P* < 10^–4^) was displayed between Ct values of IP4 sequence of SARS-CoV-2 and log_10_ starting quantity of SARS-CoV-2 RNA copies: this curve was used in each RT-qPCR serial analyses to validate our quantitative assay to check that obtained Ct values inversely reflected viral load levels (Fig. 1A).

Among 17,911 study outpatients, 496 (2.8%) patients (298 women/198 men; mean age (± SD), 32.31 ± 17.79 years) were detected positive with low to high viral loads (median (± SD) Ct value of 23.57 ± 6.74) of which 180 (36.3%) were asymptomatic. We identified two distinct subsets of viral load values that were strongly segregated by a cut-off Ct value of 30 in both asymptomatic and symptomatic patients (*P* < 10^–4^) and this result appeared to be not associated with significant differences in median age levels between these two subgroups (*P* = 0.85) (Fig. 1B). We found that the proportion of asymptomatic patients with low viral loads was significantly higher than that observed in symptomatic patients (Ct values ≥ 30: 25% versus 11.7%, *P* = 0.003, Fig. 1B). Remarkably 75% of asymptomatic patients had mean to high viral loads which median value was slightly higher than that observed in symptomatic patients (Ct values < 30: 21.60 ± 0.24 vs. 22.57 ± 0.38; *P* = 0.029) (Fig. 1B). As expected, viral RNA loads in upper respiratory tract of study outpatients appeared to be well correlated with the time delay between the respiratory sample and the onset symptoms (Fig. 1C). Only the viral loads of patients who were sampled between 8 and 28 days after symptom onset were significantly lower than those of asymptomatic patients (*P* = 0.041) (Fig. 1D).

**Figure 2:**
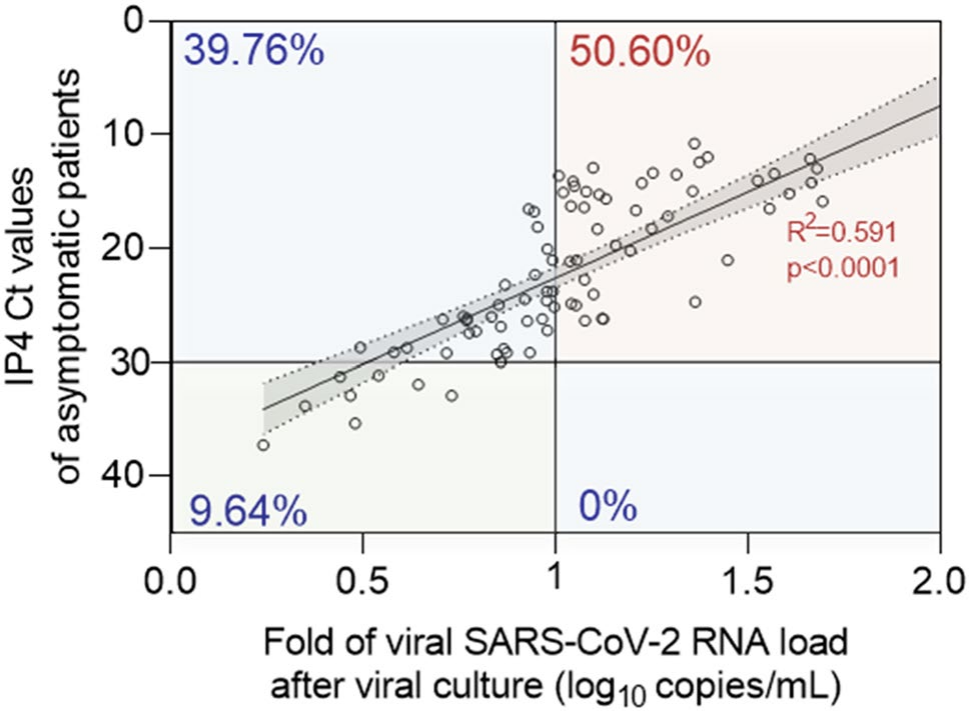
Detection of viable SARS-CoV-2 particles in upper respiratory tract of 83 COVID-19 asymptomatic adult patients. Linear regression curve between IP4 Ct values in COVID-19 asymptomatic patients and fold of viral SARS-CoV-2 RNA load level increase after 96 h viral culture on Vero cells (log10 copies/mL) (R^2^ = 0.591, *P* < 10^–4^) (n = 83). Fold values greater than 1 were considered as representative of a viral RNA replication activity related to an infection of cultured cells by viable viral particles. *Linear regression was performed, and slopes were compared using Spearman test. P values* < *0.05 were considered as statistically significant.*

To investigate whether asymptomatic patients were SARS-CoV-2 infectious shedders, we assessed the existence of a viral genomic RNA replication activity following infection of cultured cells by their upper respiratory tract specimens. Among 180 asymptomatic study patients composed of 135 subjects with RT-PCR Ct values lower than 30 and 45 subjects with RT-PCR Ct values upper than 30, only 83 had frozen stored available samples that were tested by our culture assay protocol. Results were expressed as fold of RNA viral load level increase per ml of cell culture supernatants at 96 h post-inoculation of Vero cells (Fig. 2). We observed a strong correlation (R^2^ = 0.591, *P* < 0.0001) between initial SARS-CoV-2 RT-PCR Ct values in respiratory specimens and fold increase of viral RNA levels following viral culture of the same samples (Fig. 2).

This correlation confirmed the reported association between RNA load levels and viral infectivity in upper respiratory tract of COVID-19 patients and demonstrated the reliability of our experimental approach^7–10^. Among studied asymptomatic patients, 50.6% of them were characterized by the presence of viable virus (fold of viral RNA level increase > 1) associated with RT-PCR Ct values lower than 30 in their respiratory specimens, whereas 39.76% of them evidenced no viable viral particles (fold of viral RNA level increase < 1) despite RT-PCR Ct values lower than 30 in their respiratory specimens. None of the asymptomatic patients with RT-PCR Ct values upper than 30 in their upper respiratory tract demonstrated the presence of detectable replicative viral particles (Fig. 2).

## Discussion

Few reports compared upper respiratory tract RNA loads between asymptomatic and symptomatic SARS-CoV-2 infected patients^4,5,11,12^. In the present investigation, we remarkably identify major proportions of asymptomatic adult outpatients with mean to high SARS-CoV-2 RNA loads in upper respiratory tract which median value was slightly higher than that observed in symptomatic outpatients (Fig. 1B). As recently reported in a meta-analysis compelling SARS-CoV-2, SARS-CoV, and MERS-CoV investigations, a viable viral shedding in the upper respiratory tract is present during pre-symptomatic or symptomatic phase with cultivable SARS-CoV-2 titers in the upper respiratory tract that peak in the first week of illness, whereas SARS-CoV-2 RNA shedding in respiratory samples can be prolonged during several weeks^10,13^. Two recent publications on COVID-19 patients reported no infectious virus detectable by classical cell culture assays when RT-PCR estimated by threshold cycle (Ct) values were more than 30 in the respiratory tract samples^8,14,15^. Taken together, these reports allowed us to firstly evaluate the potential infectiousness of our study COVID-19 outpatient indicating that subjects with median to high RT-PCR assays (Ct values lower than 30) may shed infectious virus in their respiratory tract and therefore may be active viral transmitters^8,14,16^ (Fig. 1).

Recent published investigations reported high viral loads in upper respiratory tract of children and elderly patients without any significantly viral load levels variations between asymptomatic, pre-symptomatic or symptomatic patients^11,12^. However, our present investigation included only young symptomatic and asymptomatic outpatients and was original by covering a large age-range (Fig. 2).

Herein, we observed the highest viral load levels in trans-nasal samples at the time or less than 4 days of symptom onset confirming that infectiousness peaked occurred on or before symptom onset^15–17^.Interestingly, our findings demonstrated that the mean RNA load value measured in asymptomatic outpatients was not statistically different from those detected in patients sampled between 5 and 7 days after the onset of symptoms indicating that they may be equivalent in terms of infectiousness (Fig. 1D). Moreover, as 75% of asymptomatic patients displayed high viral load values in their upper respiratory tract (mean Ct value (± SD) 20.08 ± 2.97**)** it is possible that an unsuspected part of this patient sub-group could be chronic respiratory tract viral shedders during several weeks and may be defined as COVID-19 super-spreaders (Fig. 1D)^18^.

In previous published reports, there is some evidence that patients may not be infectious for the entire period that they are SARS-CoV-2 positive by RT-PCR assay and that infectivity may be related to the viral load and time since symptom onset^8,19^. In the present report, our results compared RNA viral load levels between upper respiratory tract samples taken from symptomatic and asymptomatic SARS-CoV-2 infected adult outpatient subgroups (Fig. 1). In the present investigation, cultured cell isolation of virus was used the referenced method to determine contagiousness of asymptomatic patient because a positive viral RNA detection by RT-PCR did not strictly correlate with the presence of infectious virus in respiratory specimens^7,9^. Based on our cell culture assay results, we can suggest that asymptomatic patients with Ct values equal or above 30 was not an infectious viral particles shedder and thus appeared as being not contagious (Fig. 2). Our findings appear to be fully in agreement with those previously published that demonstrated a relationship of decreasing positive culture proportional to decreasing viral load and inferred that patients with threshold Ct values higher than 28–34 were no longer contagious^4,5,9,12^. Although we are unable to quantify the contributions of asymptomatic patients to transmission of SARS-CoV-2, our results evidenced that asymptomatic patients had the potential for significant infectious viral shedding with viral titers estimated from 10^1^ to 10^6^ PFU/mL of respiratory specimens (data not shown). Shedding of high viral titers (> 10^3^ PFU/mL) in the respiratory tract of COVID-19 asymptomatic patients might result in significant production of infectious droplets and possibly aerosol viral transmission in various epidemiological conditions^8,9,16^.

In conclusions, our SARS-CoV-2 RNA loads measures in upper respiratory tract samples revealed that asymptomatic outpatients shed significant levels of infectious virus in their respiratory tract playing a major role as viral transmitters in various epidemiological transmission chains, promoting COVID-19 resurgence. The impact of asymptomatic viral shedders on COVID-19 epidemic dynamics remains to be assessed in further longitudinal multicenter studies.
